# A high-coverage draft genome of the mycalesine butterfly *Bicyclus anynana*

**DOI:** 10.1093/gigascience/gix035

**Published:** 2017-05-09

**Authors:** Reuben W. Nowell, Ben Elsworth, Vicencio Oostra, Bas J. Zwaan, Christopher W. Wheat, Marjo Saastamoinen, Ilik J. Saccheri, Arjen E. van’t Hof, Bethany R. Wasik, Heidi Connahs, Muhammad L. Aslam, Sujai Kumar, Richard J. Challis, Antónia Monteiro, Paul M. Brakefield, Mark Blaxter

**Affiliations:** 1 Institute of Evolutionary Biology, University of Edinburgh, Edinburgh EH9 3FL, UK; 2 Department of Genetics, Evolution and Environment, University College London, London, UK; 3 Laboratory of Genetics, Wageningen University, Wageningen, the Netherlands; 4 Department of Zoology, Stockholm University, Stockholm, Sweden; 5 Metapopulation Research Centre, Department of Biosciences, University of Helsinki, Helsinki, Finland; 6 Institute of Integrative Biology, University of Liverpool, Liverpool L69 7ZB, UK; 7 Department of Ecology and Evolutionary Biology, Yale University, New Haven, CT 06511, USA; 8 Department of Biological Sciences, National University of Singapore, Singapore 117543; 9 Yale-NUS College, Singapore 138609; 10 Department of Zoology, University of Cambridge, Cambridge, CB2 3EJ, UK

**Keywords:** *bicyclus anynana*, squinting bush brown, nymphalidae, nymphalid, satyrid, lepidopteran genome

## Abstract

The mycalesine butterfly *Bicyclus anynana*, the “Squinting bush brown,” is a model organism in the study of lepidopteran ecology, development, and evolution. Here, we present a draft genome sequence for *B. anynana* to serve as a genomics resource for current and future studies of this important model species. Seven libraries with insert sizes ranging from 350 bp to 20 kb were constructed using DNA from an inbred female and sequenced using both Illumina and PacBio technology; 128 Gb of raw Illumina data was filtered to 124 Gb and assembled to a final size of 475 Mb (∼×260 assembly coverage). Contigs were scaffolded using mate-pair, transcriptome, and PacBio data into 10 800 sequences with an N50 of 638 kb (longest scaffold 5 Mb). The genome is comprised of 26% repetitive elements and encodes a total of 22 642 predicted protein-coding genes. Recovery of a BUSCO set of core metazoan genes was almost complete (98%). Overall, these metrics compare well with other recently published lepidopteran genomes. We report a high-quality draft genome sequence for *Bicyclus anynana*. The genome assembly and annotated gene models are available at LepBase (http://ensembl.lepbase.org/index.html).

## Data Description

**Figure 1:**
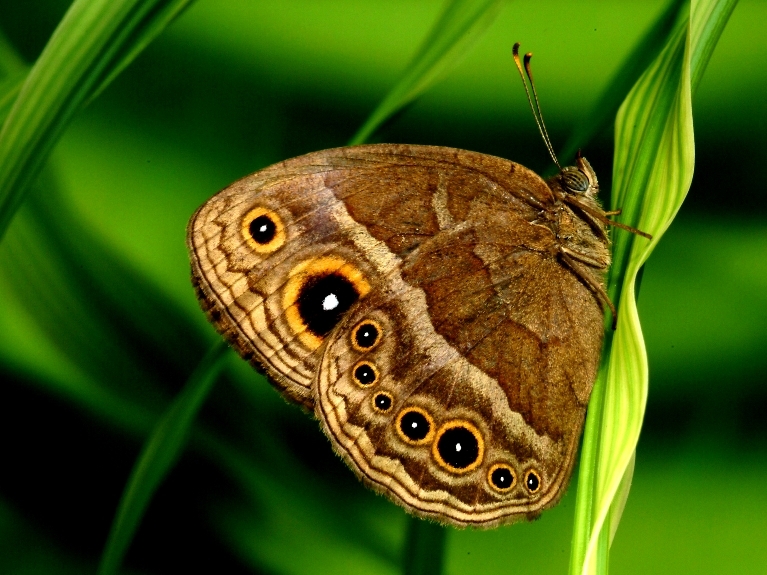
Wet-season morph of *Bicyclus anynana* (picture credit: William H. Piel and Antónia Monteiro).

The squinting bush brown butterfly, *Bicyclus anynana*, is a member of the remarkably speciose nymphalid subtribe Mycalesina, which is distributed across the Old World tropics (Fig. 1). *B. anynana* is an important model organism for the study of lepidopteran ecology, development, speciation, behaviour, and evolution [1–6]. *B. anynana* are found primarily in woodland habitats across East Africa (from southern Sudan in the north to Swaziland in the south), and adults are typically observed flying close to the ground, where[Table tbl1] they feed on fallen fruit [1]. Strikingly, *B. anynana* exhibits seasonal polyphenism, a form of phenotypic plasticity whereby individuals that develop during the wet season differ in behaviour, appearance, and life history to those that develop during the dry season [7–9]. Wet season butterflies are smaller, have shorter lifespans, are more active, and show larger and more conspicuous eyespots on their wings in comparison to dry season individuals. The genetic basis of this plasticity and its impacts on various other life history and developmental characteristics are ongoing research questions to which the availability of a *B. anynana* reference genome will contribute [10–12].

### Sampling and sequencing

Genomic DNA was extracted from a *B. anynana* female that had been inbred via 7 generations of brother-sister matings. The captive laboratory stock population from which these individuals originated was established in 1988 from 80 wild-caught individuals and has been maintained at large effective population sizes to minimise the loss of genetic diversity [1]. Two short-insert libraries with insert sizes of 350 and 550 bp were constructed using Illumina TruSeq Nano reagents and sequenced (125 base, paired-end) on an Illumina HiSeq2500 at Edinburgh Genomics (Edinburgh, UK). DNA from a sister to this focal animal was used to construct four long-insert (mate-pair) libraries with insert sizes of 3 and 5 kb (2 of each) at the Centre for Genomic Research, University of Liverpool (Liverpool, UK); libraries of both insert-sizes were then sequenced on an Illumina HiSeq2500 and an Illumina MiSeq at Edinburgh Genomics (Table 1). DNA from a female descendent of the same inbred line was used to construct 2 long read libraries with insert sizes of 10 and 20 kb, sequenced on the PacBio platform at the Genome Institute of Singapore at ∼×10 coverage using 16 P6 SMRT cells. All raw data have been deposited in the Short Read Archive under the accessions given in Table 1.

**Table 1: tbl1:** Data counts and library information.

Library type	Platform	Read length	Insert size (expected)	Number of reads (raw)	Number of reads (trimmed)	Number of bases (trimmed)	SRA run accessions
Short insert	Illumina HiSeq2500	125 bp paired-end	350 bp	271 808 057 pairs	267 241 712 (98.3%)	66 334 099 834 (97.6%)	ERR1102671-2, ERR1102675-6
Short insert	Illumina HiSeq2500	125 bp paired-end	550 bp	241 050 065 pairs	234 269 871 (97.2%)	57 913 474 128 (96.1%)	ERR1102673-4, ERR1102677-8
Mate pair	Illumina HiSeq2500	100 bp paired-end	3 kb	77 105 680 pairs	31 848 200 (41.3%)	5 758 856 502 (37.3%)	ERR1750945
Mate pair	Illumina MiSeq	100 bp paired-end	3 kb	5 641 764 pairs	2 170 610 (38.5%)	397 993 018 (35.3%)	ERR754051
Mate pair	Illumina HiSeq2500	100 bp paired-end	5 kb	77 614 870 pairs	45 676 725 (58.9%)	8 203 769 131 (52.8%)	ERR1750946
Mate pair	Illumina MiSeq	100 bp paired-end	5 kb	7 939 601 pairs	4 734 000 (59.6%)	861 352 793 (54.2%)	ERR754052
Long read	PacBio P6	0.80–50 kb	10 kb	1 388 796	1 199 064 (86.3%)	4 086 394 966	ERR1797559-74

**Figure 2:**
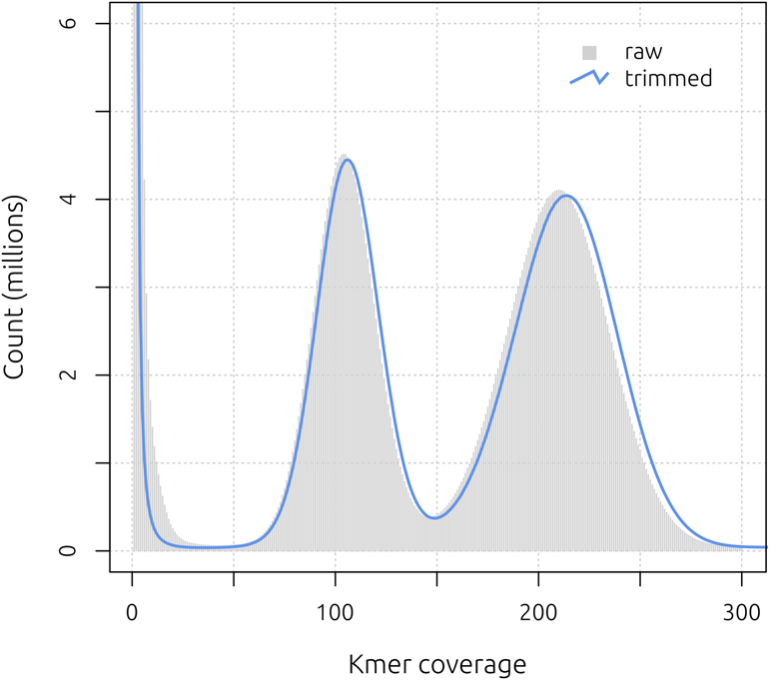
Kmer frequency distribution for *B. anynana* short-insert libraries (*k* = 31). The bimodality of the distribution, with peaks at approximately ×105 and ×210, is the result of heterozygosity in the sequence data.

**Figure 3: fig3:**
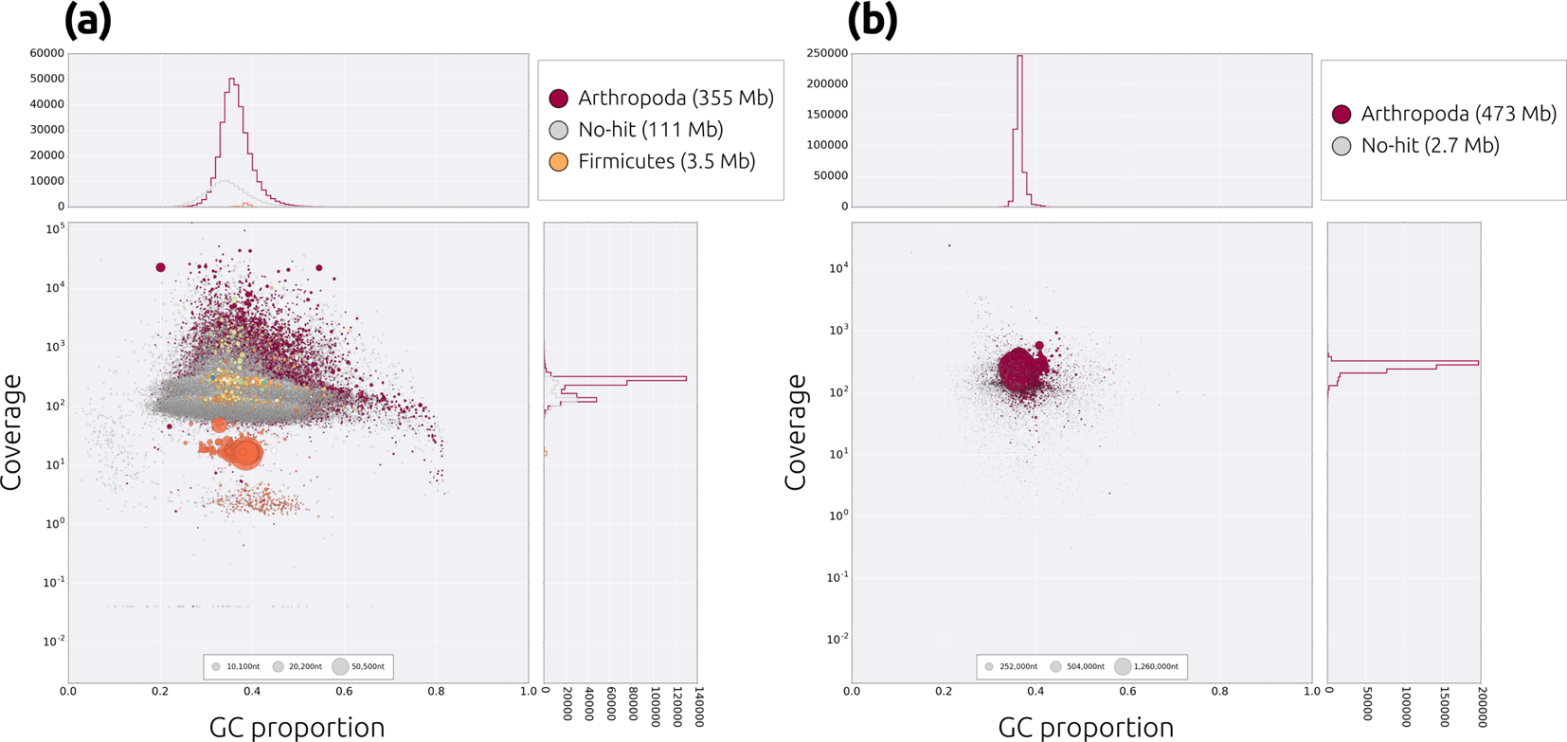
Taxon-annotated GC-coverage plots for **(a)** draft and **(b)** final *B. anynana* genome assemblies. Each contig/scaffold in the assembly is represented by a circle, coloured according to the best match to taxonomically annotated sequence databases (see legends) and distributed according to the proportion GC (*x*-axis) and read coverage (*y*-axis). The upper- and right-hand panels show the distribution of the total span (kb) of contigs/scaffolds for a given coverage (upper panel) or GC (right panel) bin. The heterozygosity in the sample is evident in the bimodal coverage distribution seen in (a). The cluster of orange-coloured contigs at a lower coverage and higher GC than the main cloud were likely derived from contaminant *Enterococcus* present in the sample. The final assembly (b) shows the effective collapse of heterozygous regions, the removal of contaminant sequences, and the scaffolding of contigs into long contiguous sequences. Note that only taxon annotations with a span > 1 Mb are shown in the legend for clarity.

A total of 128.2 Gb of raw Illumina data was filtered for low-quality bases and adapter contamination using Skewer v. 0.2.2 [13], and both raw and trimmed reads were inspected using FastQC v. 0.11.4 [14]. Only 4 Gb of data (3.1%) was discarded, indicating the high quality of the raw data. Kmer frequency distributions were estimated using the “kmercountexact” program from the BBMap v. 36.02 package [15] and showed 2 major coverage peaks at ∼×105 and ∼×210 (Fig. 2). The first peak (×105) represents the proportion of the genome that is heterozygous and has an approximate span of 87.7 Mb (18.4% of the genome; calculated[Fig fig3] as one-half of the area under the ×105 curve, from ×50 to ×150). The expected proportion of heterozygous sites given 7 brother-sister (full-sib) matings is 0.75^⁁^7 = 13.3%, or 63.5 Mb. Thus, the greater than expected heterozygosity is likely to be due primarily to selection against highly inbred individuals during the course of the inbreeding regime [16].

### Contaminant filtering and assembly

Short-insert libraries were screened for the presence of contaminant reads using Taxon-Annotated GC-Coverage (TAGC) plots, or “blobplots” [17]. An initial draft assembly was constructed using the CLC assembler (CLCBio, Copenhagen) and compared to the NCBI nucleotide database (nt) using Megablast v. 2.3.0+ [18], and against the UniRef90 protein database using Diamond v. 0.7.10 [19]. Read coverage for each contig was calculated by mapping both libraries to the CLC assembly using CLC mapper (CLCBio, Copenhagen), and blobplots were generated using Blobtools v. 0.9.19.4 [20] using the “bestsumorder” rule for taxonomic annotation of contigs (Fig. 3). Contigs that showed a substantially different coverage relative to that of the main cluster of contigs and/or good hits to sequences annotated as non-Arthropoda were classed as putative contaminants. A total of 237 394 pairs of reads (∼59 Mb) that were classed as either “mapped/mapped” or “mapped/unmapped” to a putative contaminant were subsequently discarded from further analysis.

Filtered libraries were reassembled using the heterozygous-aware assembler Platanus v. 1.2.4 [21], with default parameters. Contigs were further scaffolded with the mate pair libraries using SSPACE v. 3.0 [22] and with 35 747 assembled *B. anynana* transcripts [23] using a combination of L_RNA_scaffolder [24] and SCUBAT v. 2 [25]. A final round of scaffolding was performed with PacBio long reads (fastq files error-corrected using the RS_Preaassembler.2 protocol) using SSPACE-LongRead v. 1.1 [26]. Finally, gaps between scaffolds were filled using GapFiller v. 1.10 [27] and PBJelly v. 15.8.24 [28].

Our final assembly (v. 1.2) comprised 10 800 scaffolds spanning a total of 475.4 Mb, with a scaffold N50 of 638 kb (Table 2). The genome-wide proportion of G+C was 36.5%, while the number of undetermined bases (Ns) was 5.8 Mb (∼1.2% of the total span). We determined assembly completeness by mapping both genomic and transcriptomic reads from *B. anynana* (SRA whole genome sequencing accessions ERR1102671-8 and transcriptome accessions ERR1022636-7, ERR1022640-1, and ERR1022644-5, downloaded October 2016) to the genome using BWA mem v. 0.7.12 [29] and STAR v. 020201 [30], respectively. Over 99% of reads from the 2 short-insert libraries mapped to the assembly, suggesting that the vast majority of the genome represented by these data has been assembled. In addition, 94.9% of RNA-Seq reads mapped to the assembly, suggesting that the majority of transcribed genes are present. Gene-level completeness was assessed using CEGMA v. 2.5 [31] and BUSCO v. 2.0 [32]. The proportion of CEGMA genes “completely” recovered (*n* = 248) was 81%, increasing to 97% when partially recovered genes were included. The recovery of BUSCO genes specific to the metazoa (*n* = 978) was higher, at 98% for complete genes, increasing to 99% when partial genes were included. An almost complete set (99.2%) of BUSCO genes specific to the Arthropoda (*n* = 1066) was also recovered. In addition, CEGMA indicated a duplication rate of 1.1 while BUSCO estimated only ∼2% of genes were present in multiple copies. The high complete CEGMA/BUSCO scores suggestthat a good assembly has captured the majority of core metazoan/Arthropod genes in full length and that the fragmentation of genes across multiple scaffolds is low. In addition, the low duplication rates suggest that most genes are present in single copy, and thus that the genome does not include significant duplicated segments representing alternative haplotypes.

**Table 2:** Summary of *B. anynana* genome assembly and comparison to selected lepidopteran genomes.

	*B. anynana*	*B. mori*	*D. plexippus*	*H. melpomene*	*M. cinxia*
Assembly version	1.2	ASM15162v1	3	Hmel2	MelCinx1.0
Span	475.4 Mb	481.8 Mb	248.6 Mb	275.2 Mb	389.9 Mb
Contigs					
Number	23 699	88 673	10 682	3100	48 180
N50^a^	78.7 kb	15.5 kb	111.0 kb	328.9 kb	14.1 kb
NumN50^b^	1543	8075	548	214	7366
Scaffolds					
Number	10 800	43 379	5397	795	8261
N50	638.3 kb	4008.4 kb	715.6 kb	2102.7 kb	119.3 kb
NumN50	194	38	101	34	970
N90	99.3 kb	61.1 kb	160.5 kb	273.1 kb	29.6 kb
NumN90	909	258	366	176	3396
Shortest/longest	201 b/5 Mb	53 b/16.2 Mb	300 b/6.2 Mb	394 b/9.4 Mb	1.5 kb/668 kb
G+C content	36.5%	37.7%	31.6%	32.8%	32.6%
NNNs					
Span	5.8 Mb (1.2%)	50.1 Mb (10.4%)	6.7 Mb (2.7%)	986 kb (0.4%)	28.9 Mb (7.4%)
N50	1.4 kb	5.0 kb	2.5 kb	2.4 kb	1.4 kb
CEGMA^c^ (*n* = 248)	C: 81.1%; D: 1.1; F: 97.2%	C: 76.6%; F: 96.8%	C: 90.3%; F: 96%	C: 88.7%; F: 96.8%	NA
BUSCO^c^ (*n* = 1066)	C: 98.3%; D: 1%; F: 99.2%	C: 97.5%; D: 0.5%; F: 98.4%	C: 97.4%; D: 8.6%; F: 98.5%	C: 98.8%; D: 0.7%; F: 99.3%	C: 85.7%; D: 0.2%; F: 91.8%

^a^N50: the length of the contig/scaffold at which 50% of the genome span is accounted for, given a list of sequences sorted by length. ^b^numN50: the number of sequences required to reach the N50 sequence. ^c^CEGMA/BUSCO notation: C, proportion (%) of genes completely recovered; D, duplication rate; F, proportion (%) of genes at least partially recovered (including complete genes); *n*, number of queries. Note that duplication rate (D) for CEGMA is given as the average number of (complete) genes recovered, whereas for BUSCO it is the proportion of complete genes recovered multiple times. BUSCO values are based on comparisons to the Arthropoda gene set.

### Annotation

Prior to gene prediction, we masked the *B. anynana* assembly for repetitive elements to minimise the number of spurious open-reading frames due to low-complexity repeat regions or transposable elements. Repetitive motifs in the *B. anynana* assembly were modelled *ab initio* using RepeatModeler v. 1.0.5 (http://www.repeatmasker.org/RepeatModeler.html). Repeats occurring within genuine coding regions were excluded by querying the proteins from a previous *B. anynana* assembly (v. 0.1) versus the RepeatModeler database using BLAST, removing any sequences showing a match at the *E*-value ≤ 1e-10 threshold. The filtered RepeatModeler database was combined with known repeats from the Lepidoptera using RepBase v. 20.05 [33] and input to RepeatMasker v. 4.0.5 [34] to mask the assembly. Overall, approximately one-quarter of the assembly (122.6 Mb) was masked from gene prediction (Table 3).

**Table 3:** Major types of repeat content for *B. anynana*.

Repeat type	Span (Mb)	Proportion of genome
SINE	10.8	2.3%
LINE	15.3	3.2%
LTR elements	1.1	0.2%
DNA elements	0.8	0.2%
Small RNA	10.8	2.3%
Unclassified	86.2	18.1%
Total	122.6	25.8%

Gene finding was performed following a 2-pass approach [35]. Initial gene models were constructed with MAKER v. 2.31 [36] using HMMs derived from SNAP [37] and GeneMark-ES v. 4.3 [38] in conjunction with a recently published *B. anynana* transcriptome as evidence. MAKER gene models were then passed to AUGUSTUS v. 3.0.3 [39] for refinement, resulting in an initial set of 26 722 predicted protein-coding genes. A set of basic filters was applied to remove likely spurious gene models (Table 4), resulting in the deletion of 4080 gene models. Protein sequences from the filtered 22 642 genes were annotated using BLAST searches versus UniRef90 and the NCBI non-redundant protein database (nr), and domains/motifs were described using InterProScan5 [40]. Summary statistics for the 22 642 predicted gene models are given in Table 5.

**Table 4:** Number of genes in potential error categories.

Category	Description	Number of genes
(a)	Single-exon	7112
(b)	Small exon (<9bp)	1866
(c)	Small intron (≤40 bp)	45
(d)	Short (CDS < 120 bp)	127
(e)	No hit to *nr*	6532
(f)	Duplicate (≥98% identity over ≥98% query length)	822
Total^a^		4080

^a^Defined as the non-redundant total of the intersection of each category (a) to (d) with category (e), plus the shorter of any duplicates identified in category (f).

**Table 5:** Summary of *B. anynana* gene prediction.

	*B. anynana*	*B. mori*	*D. plexippus*	*H. melpomene*	*M. cinxia*
Assembly version	1.2	ASM15162v1	3	Hmel2	MelCinx1.0
Number of CDS	22 642	19 618	15 130	13 178	16 668
Mean length	1.4 kb	1.6 kb	1.4 kb	1.3 kb	958 bp
Median length	1.2 kb	1.2 kb	981 bp	927 bp	693 bp
Min/max	84 bp/28.3 kb	23 bp/60.3 kb	9 bp/58.9 kb	45 bp/46.4 kb	6 bp/45.4 kb
Introns					
Mean number per gene	4.4	9.9	5.7	5	NA^a^
Length (mean/median)	1.3/0.6 kb	2.4/0.8 kb	795/280 bp	960/416 bp	NA
Exons					
Length (mean/median)	208/126 bp	283/161 bp	206/149 bp	284/157 bp	NA
Number of single-exon genes	3571	1744	1461	3113	NA
Transcript GC	49.2%	48.3%	46.5%	43%	41.7%
Gene frequency^b^ (genes per Mb)	47.7	32.1	60.9	55.5	NA

^a^GFF for *M. cinxia* not available. ^b^Defined as the number of genes divided by the total genome span (Mb).

### Comparison to other lepidopteran genomes

**Figure 4:**
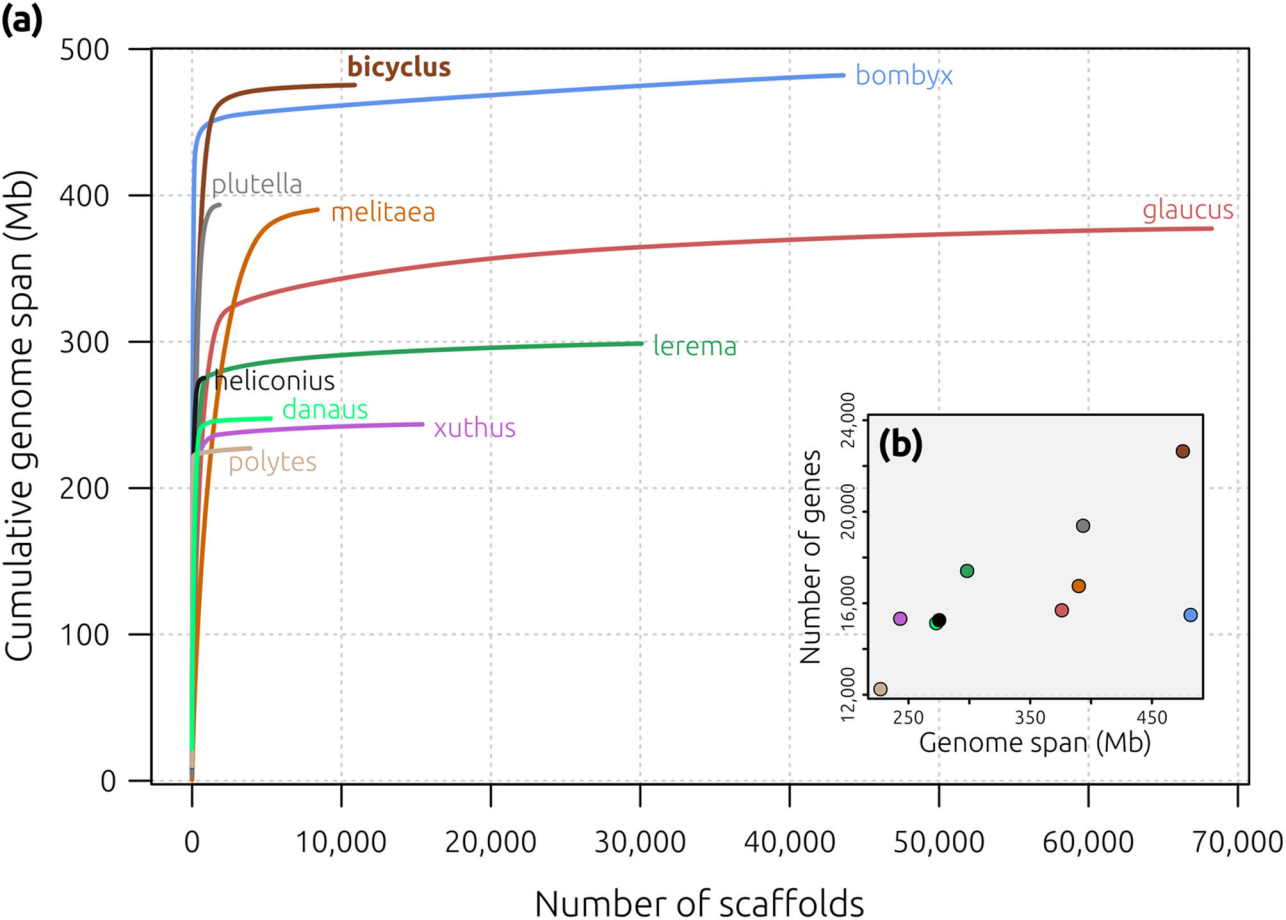
Assembly and gene prediction comparison among 10 lepidopteran genomes. **(a)** Cumulative assembly curves showing the relationship between the number of scaffolds (*x*-axis) and the cumulative span of each assembly (*y*-axis), coloured by species. Higher-quality assemblies are represented by an almost-vertical line (e.g., *H. melpomene* Hmel2 assembly in black), indicating that a relatively small number of scaffolds is required to reach the final genome span; conversely, a long tail indicates that the assembly includes a large number of smaller scaffolds. The curve for *B. anynana* (brown and bold) suggests a good assembly for this species, with the majority of the assembly comprised of relatively few scaffolds. **(b)***B. anynana* v. 1.2 encodes the greatest number of genes of the 10 genomes and is particularly different from *B. mori*, which is of equivalent length. Species names/colours are as follows: “bicyclus” (brown), *B. anynana*; “bombyx” (blue), *B. mori*; “danaus” (light green), *D. plexippus*; “heliconius” (black), *H. melpomene*; “lerema” (dark green), *L. accius*; “melitaea” (orange), *M. cinxia*; “glaucus” (red), *P. glaucus*; “polytes” (pink), *P. polytes*; “xuthus” (violet), *P. xuthus*; “plutella” (grey), *P. xylostella*.

To ascertain the relative quality of the *B. anynana* v. 1.2 assembly, we compared our results to 9 other published lepidopteran genomes available on LepBase (http://lepbase.org/) [41]: *Bombyx mori* ASM15162 v. 1 [42], *Danaus plexippus* v. 3 [43], *Heliconius melpomene* Hmel2 [44,45], *Lerema accius* v. 1.1 [46], *Melitaea cinxia* MelCinx1.0 [47], *Papilio glaucus* v. 1.1 [48], *Papilio polytes* Ppol 1.0 [49], *Papilio xuthus* Pap_xu_1.0 [49], and *Plutella xylostella* DBM_FJ_v1.1 [50]. The *B. anynana* v. 1.2 assembly was of high quality compared to other published genomes, with the majority of the genome represented in a relatively small number of scaffolds despite being only marginally smaller than the largest lepidopteran genome, *B. mori* (Fig. 4a). Interestingly, *B. anynana* v. 1.2 encodes the highest number of proteins of the 10 species compared (Fig. 4b). Despite measures to eliminate potentially spurious ORFs caused by annotation error or by duplication, *B. anynana* encodes ∼3250 more genes than the diamondback moth *P. xylostella*, and ∼10 400 more than the swallowtail *P. polytes*. It is tempting to attribute the apparently high number of genes to the developmental plasticity and alternative seasonal forms with divergent morphologies and life histories in *B. anynana*. However, it remains to be determined whether the number of genes predicted in *B. anynana* is a function of its larger genome size or unusual life history characteristics, or if further curation of the v. 1.2 gene models will reduce the number of inferred genes.

### Concluding remarks

We present a high-coverage, high-quality draft assembly and annotation of the mycalesine butterfly *B. anynana*. The assembly will be a core resource for ongoing analyses of population genomics, discovery of *cis*-regulatory elements of wing patterning and other genes, functional genetics and functional ecology of complex gene families, and the evolution of novel and plastic lifecycle strategies in lepidopterans and other arthropods.

### Abbreviations

BUSCO: Benchmarking Universal Single-Copy Orthologs; CEGMA: Core Eukaryotic Genes Mapping Approach; CDS: coding sequence; ORF: open reading frame.

## Supplementary Material

GIGA-D-17-00016_Original_Submission.pdf

GIGA-D-17-00016_Revision_1.pdf

Response_to_Reviewers_Original_Submission.pdf

Reviewer_1_Report_(Original_Submission).pdf

Reviewer_2_Report_(Original_Submission).pdf

## Availability of supporting data

All raw sequence data have been deposited in the Short Read Archive (SRA) and are available for download using the accession numbers provided in Table 1. The *B. anynana* v. 1.2 assembly, as well as final predicted gene models and protein annotations, are publicly available for viewing and download via LepBase [41], an Ensembl [51] genome database for the Lepidoptera (http://ensembl.lepbase.org/index.html). Data supporting the manuscript, including annotations as well as BUSCO and CEGMA results, are also available via the *GigaScience* database, GigaDB [52]. A previous *B. anynana* assembly (nBa.0.1) is also available on LepBase.
