# Supplementary material for: A high-coverage draft genome of the mycalesine butterfly Bicyclus anynana
Source: Gigascience. 2017 May 9;6(7):1–7. doi: 10.1093/gigascience/gix035 (PMC5493746; doi:10.1093/gigascience/gix035)

A high-coverage draft genome of the mycalesine butterfly *Bicyclus anynana*

Reuben W. Nowell[1,*], Ben Elsworth[1], Vicencio Oostra[2], Bas J. Zwaan[3], Christopher W. Wheat[4], Marjo Saastamoinen[5], Ilik J. Saccheri[6], Arjen E. van't Hof[6], Bethany R. Wasik[7], Heidi Connahs[8], Muhammad L. Aslam[8], Sujai Kumar[1], Richard J. Challis[1], Antónia Monteiro[7,8,9], Paul M. Brakefield[10] and Mark Blaxter[1,*]

*Correspondence: reubennowell@gmail.com; mark.blaxter@ed.ac.uk

Abstract

Background: The mycalesine butterfly *Bicyclus anynana*, the 'Squinting bush brown', is a model organism in the study of lepidopteran ecology, development and evolution. Here, we present a draft genome sequence for *B. anynana* to serve as a genomics resource for current and future studies of this important model species.

Findings: Eight libraries with insert sizes ranging from 350 bp to 20 kb were constructed using DNA from an inbred female and sequenced using both Illumina and PacBio technology. 128 Gb raw Illumina data were filtered to 124 Gb and assembled to a final size of 475 Mb (~260X assembly coverage). Contigs were scaffolded using mate-pair, transcriptome and PacBio data into 10,800 sequences with an N50 of 638 kb (longest scaffold 5 Mb). The genome is comprised of 26% repetitive elements, and encodes a total of 22,642 predicted protein-coding genes.

Recovery of a BUSCO set of core metazoan genes was almost complete (98%). Overall, these metrics compare well with other recently published lepidopteran genomes.

Conclusions: We report a high-quality draft genome sequence for *Bicyclus anynana*. The genome assembly and annotated gene models are available at LepBase (http://ensembl.lepbase.org/index.html).

Keywords: *Bicyclus anynana*, Squinting bush brown, Nymphalidae, nymphalid, satyrid, lepidopteran genome.

Data description

The squinting bush brown butterfly, *Bicyclus anynana*, is a member of the remarkably speciose nymphalid subtribe Mycalesina, which is distributed across the Old World tropics. *B. anynana* is an important model organism for the study of lepidopteran ecology, development, speciation, behaviour, and evolution [1-6]. *B. anynana* are found primarily in woodland habitats across East Africa (from southern Sudan in the north to Swaziland in the south), and adults are typically observed flying close to the ground where they feed on fallen fruit [1]. Strikingly, *B. anynana* exhibits seasonal polyphenism, a form of phenotypic plasticity whereby individuals that develop during the wet season differ in both behaviour, appearance and life history to those that develop during the dry season [7-9]. Wet season butterflies are smaller, have shorter lifespans, are more active, and show larger and more conspicuous eyespots on their wings in comparison to dry season individuals. The genetic basis of this plasticity, and its impacts on various other life-history and developmental

characteristics, are ongoing research questions to which the availability of a *B. anynana* reference genome will contribute [10-12].

Sampling and sequencing

Genomic DNA was extracted from a *B. anynana* female that had been inbred via seven generations of brother-sister matings. The captive laboratory stock population from which these individuals originated was established in 1988 from 80 wild-caught individuals, and has been maintained at large effective population sizes to minimise the loss of genetic diversity [1]. Two short-insert libraries with insert sizes of 350 and 550 bp were constructed using Illumina TruSeq Nano reagents and sequenced (125 base, paired end) on an Illumina HiSeq2500 at Edinburgh Genomics (Edinburgh, UK). DNA from a sister to this focal animal was used to construct four long-insert (mate-pair) libraries with insert sizes of 3 and 5 kb (two of each) at the Centre for Genomic Research, University of Liverpool (Liverpool, UK); libraries of both insert-sizes were then sequenced on an Illumina HiSeq2500 and an Illumina MiSeq at Edinburgh Genomics (Table 1). DNA from a female descendent of the same inbred line was used to construct two long read libraries with insert sizes of 10 and 20 kb, sequenced on the PacBio platform at the Genome Institute of Singapore at 20x coverage using 16 P6 SMRT cells. All raw data have been deposited in the Short Read Archive under the accessions given in Table 1.

A total of 128.2 Gb raw Illumina data were filtered for low-quality bases and adapter contamination using Skewer v0.2.2 [13], and both raw and trimmed reads were inspected using FastQC v0.11.4 [14]. Only 4 Gb data (3.1%) were discarded, indicating the high quality of the raw data. Kmer frequency distributions were

estimated using the "kmercountexact" program from the BBMap v36.02 package [15], and showed two major coverage peaks at ~105X and ~210X (Figure 1). The first peak (105X) represents the proportion of the genome that is heterozygous, and has an approximate span of 87.7 Mb (18.4% of the genome; calculated as one half of the area under the 105X curve, from 50X to 150X). The expected proportion of heterozygous sites given seven brother-sister (full-sib) matings is 0.75^7 = 13.3%, or 63.5 Mb. Thus, the greater than expected heterozygosity is likely to be due primarily to selection against highly inbred individuals during the course of the inbreeding regime [16].

Contaminant filtering and assembly

Short-insert libraries were screened for the presence of contaminant reads using Taxon-Annotated GC-Coverage (TAGC) plots, or "blobplots" [17]. An initial draft assembly was constructed using CLC assembler (CLCBio, Copenhagen) and compared to the NCBI nucleotide database (nt) using Megablast v2.3.0+ [18], and against the UniRef90 protein database using Diamond v0.7.10 [19]. Read coverage for each contig was calculated by mapping both libraries to the CLC assembly using CLC mapper (CLCBio, Copenhagen), and blobplots were generated using Blobtools v0.9.19.4 [20] using the "bestsumorder" rule for taxonomic annotation of contigs (Figure 2). Contigs that showed a substantially different coverage relative to that of the main cluster of contigs and/or good hits to sequences annotated as non-Arthropoda were classed as putative contaminants. A total of 237,394 pairs of reads (~59 Mb) that were classed as either "mapped/mapped" or "mapped/unmapped" to a putative contaminant were subsequently discarded from further analysis.

Filtered libraries were reassembled using the heterozygous-aware assembler Platanus v1.2.4 [21], with default parameters. Contigs were further scaffolded with the mate pair libraries using SSPACE v3.0 [22] and with 35,747 assembled *B. anynana* transcripts using a combination of L_RNA_scaffolder [23] and SCUBAT v2 [24]. The transcripts were assembled using Trinity v. 20140717 [25] from ca. 2 x 10^9 paired end RNA-Seq reads sequenced from thorax and abdomen tissue of 72 outbred *B. anynana* females of the standard captive laboratory stock population (Oostra et al., in preparation). A final round of scaffolding was performed with PacBio long reads (fastq files error-corrected using the RS_Preaassembler.2 protocol) using SSPACE-LongRead v1.1 [26]. Finally, gaps between scaffolds were filled using GapFiller v1.10 [27] and PBJelly v15.8.24 [28].

Our final assembly (v1.2) comprised 10,800 scaffolds spanning a total of 475.4 Mb, with a scaffold N50 of 638 kb (Table 2). The genome-wide proportion of G+C was 36.5%, while the number of undetermined bases (N's) was 5.8 Mb (~1.2% of the total span). We determined assembly completeness by mapping both genomic and transcriptomic reads from *B. anynana* (SRA whole genome sequencing accessions ERR1102671-8, and transcriptome accessions ERR1022631, ERR1022635-7, ERR1022640 and ERR1022644, downloaded October 2016) to the genome using BWA mem v0.7.12 [29] and STAR v020201 [30] respectively. Over 99% of reads from the two short-insert libraries mapped to the assembly, suggesting that the vast majority of the genome represented by these data has been assembled. In addition, 94.9% of RNA-Seq reads mapped to the assembly, suggesting that the majority of transcribed genes are present. Gene-level completeness was assessed using CEGMA v2.5 [31] and

BUSCO v2.0 [32]. The proportion of CEGMA genes "completely" recovered (*n* = 248) was 81%, increasing to 97% when partially recovered genes are included. The recovery of BUSCO genes specific to the metazoa (*n* = 978) was higher, at 98% for complete genes, increasing to 99% when partial genes are included. An almost complete set (99.2%) of BUSCO genes specific to the Arthropoda (*n* = 1,066) was also recovered. In addition, CEGMA indicated a duplication rate of 1.1 while BUSCO estimated only ~2% genes were present in multiple copies. The high complete CEGMA/BUSCO scores suggest a good assembly that has captured the majority of core metazoan/Arthropod genes in full-length, and that the fragmentation of genes across multiple scaffolds is low. In addition, the low duplication rates suggest that most genes are present in single copy, and thus that the genome does not include significant duplicated segments representing alternative haplotypes.

Annotation

Prior to gene prediction, we masked the *B. anynana* assembly for repetitive elements to minimise the number of spurious open-reading frames due to low-complexity repeat regions or transposable elements. Repetitive motifs in the *B. anynana* assembly were modelled *ab initio* using RepeatModeler v1.0.5 (http://www.repeatmasker.org/RepeatModeler.html). Repeats occurring within genuine coding regions were excluded by querying the proteins from a previous *B. anynana* assembly (v0.1) versus the RepeatModeler database using BLAST, removing any sequences showing a match at *E*-value ≤ 1e-10 threshold. The filtered RepeatModeler database was combined with known repeats from the Lepidoptera using RepBase v20.05 [33] and input to RepeatMasker v4.0.5 [34] to mask the

assembly. Overall, approximately one quarter of the assembly (122.6 Mb) was masked from gene prediction (Table 3).

Table 3: Major types of repeat content for *B. anynana*.

Repeat type	Span (Mb)	Proportion of genome
SINE	10.8	2.3%
LINE	15.3	3.2%
LTR elements	1.1	0.2%
DNA elements	0.8	0.2%
Small RNA	10.8	2.3%
Unclassified	86.2	18.1%
**Total**	122.6	25.8%

Gene finding was performed following a two-pass approach [35]. Initial gene-models were constructed with MAKER v2.31 [36], using HMMs derived from SNAP [37] and GeneMark-ES v4.3 [38] in conjunction with a set of assembled *B. anynana* transcripts as evidence (Oostra et al., in preparation). MAKER gene-models were then passed to AUGUSTUS v3.0.3 [39] for refinement, resulting in an initial set of 26,722 predicted protein-coding genes. A set of basic filters was applied to remove likely spurious gene models (Table 4), resulting in the deletion of 4,080 gene models. Protein sequences from the filtered 22,642 genes were annotated using BLAST searches versus UniRef90 and the NCBI non-redundant protein database (nr), and domains/motifs were described using InterProScan5 [40]. Summary statistics for the 22,642 predicted gene models are given in Table 5.

Table 4: Number of genes in potential error categories.

Category	Description	Number of genes
**(a)**	Single-exon	7,112
**(b)**	Small exon (< 9 bp)	1,866
**(c)**	Small intron (≤ 40 bp)	45
**(d)**	Short (CDS < 120 bp)	127
**(e)**	No hit to *nr*	6,532
**(f)**	Duplicate (≥ 98% identity over ≥ 98% query length)	822
**Total[1]**		4,080

[1]Defined as the non-redundant total of the intersection of each category (a) to (d) with category (e), plus the shorter of any duplicates identified in category (f).

Comparison to other lepidopteran genomes

To ascertain the relative quality of the *B. anynana* v1.2 assembly, we compared our results to nine other published lepidopteran genomes available on LepBase (http://lepbase.org/) [41]: *Bombyx mori* ASM15162v1 [42], *Danaus plexippus* v3 [43], *Heliconius melpomene* Hmel2 [44,45], *Lerema accius* v1.1 [46], *Melitaea cinxia* MelCinx1.0 [47], *Papilio glaucus* v1.1 [48], *Papilio polytes* Ppol 1.0 [49], *Papilio xuthus* Pap_xu_1.0 [49] and *Plutella xylostella* DBM_FJ_v1.1 [50]. The *B. anynana* v1.2 assembly was of high quality compared to other published genomes, with the majority of the genome represented in a relatively small number of scaffolds despite being only marginally smaller than the longest lepidopteran genome, *B. mori* (Figure 3a). Interestingly, *B. anynana* v1.2 encodes the highest number of proteins of the 10 species compared (Figure 3b). Despite measures to eliminate potentially spurious ORFs caused by annotation error or by duplication, *B. anynana* encodes ~3,250 more

genes that the diamondback moth *P. xylostella*, and ~10,400 more than the swallowtail *P. polytes*. It is tempting to attribute the apparently high number of genes to the developmental plasticity and alternative seasonal forms with divergent morphologies and life histories in *B. anynana*. However, it remains to be determined whether the number of genes predicted in *B. anynana* is a function of its larger genome size or unusual life-history characteristics, or if further curation of the v1.2 gene models will reduce the number of inferred genes.

Concluding remarks

We present a high-coverage, high quality draft assembly and annotation of the mycalesine butterfly *B. anynana*. The assembly will be a core resource for ongoing analyses of population genomics, discovery of *cis*-regulatory elements of wing patterning and other genes, functional genetics and functional ecology of complex gene families, and the evolution of novel and plastic lifecycle strategies in lepidopterans and other arthropods.

Availability of supporting data

All raw sequence data have been deposited in the Short Read Archive (SRA) and are available for download using the accession numbers provided in Table 1. The *B. anynana* v1.2 assembly, as well as final predicted gene-models and protein annotations, are publicly available for viewing and download via LepBase [41], an Ensembl [51] genome database for the Lepidoptera (http://ensembl.lepbase.org/index.html). A previous *B. anynana* assembly (nBa.0.1) is also available on LepBase.

Abbreviations

Gb: Gigabase; Mb: Megabase; kb: kilobase; bp: base pairs; CEGMA: Core Eukaryotic Genes Mapping Approach; BUSCO: Benchmarking Universal Single-Copy Orthologs; ORF: Open reading frame; CDS: Coding sequence

Competing interests

The authors declare that they have no competing interests.

Author contributions

PMB and MB designed the study; AM and BRW collected samples and produced the inbred line; AEVH, IJS and HC extracted DNA samples; RWN, BE and MB worked on the genome assembly and annotation; VO, BJZ, CW and MS contributed transcriptome data; AM, HC and MLA contributed PacBio data; SK and RJC uploaded the assembly to LepBase. RWN, VO, AM, PMB and MB wrote the manuscript. All authors have read and approved the final version of the manuscript.

Acknowledgements

We thank Edinburgh Genomics and Genome Institute of Singapore for genome sequencing, initial QC and data delivery. Funding for the *Bicyclus anynana* genome project was provided by the ERC Advanced Grant number 250325 (EMARES) to PMB and by the South East Asian Biodiversity Genomics Center (NUS grant R-154-000-

648-646 and R-154-000-648-733) to AM. Funding for LepBase was provided by BBSRC grant number BB/K020161.

Author details

[1]Institute of Evolutionary Biology, University of Edinburgh, Edinburgh EH9 3FL, United Kingdom; [2]Department of Genetics, Evolution and Environment, University College London, United Kingdom; [3]Laboratory of Genetics, Wageningen University, The Netherlands; [4]Department of Zoology, Stockholm University, Sweden; [5]Metapopulation Research Centre, Department of Biosciences, University of Helsinki, Finland; [6]Institute of Integrative Biology, University of Liverpool, Liverpool L69 7ZB, United Kingdom; [7]Department of Ecology and Evolutionary Biology, Yale University, New Haven, CT 06511, USA; [8]Department of Biological Sciences, National University of Singapore, Singapore 117543; [9]Yale-NUS College, Singapore 138609; [10]Department of Zoology, University of Cambridge, Cambridge, CB2 3EJ, United Kingdom

Tables

Tables 1, 2 and 5 are in landscape orientation and can be found as additional files at the end of this manuscript.

Figure legends

Figure 1: Kmer frequency distribution for *B. anynana* short-insert libraries (*k* = 31). The bimodality of the distribution, with peaks at approximately 105X and again at 210X, is the result of heterozygosity in the sequence data.

Figure 2: Taxon-annotated GC-coverage plots for **(a)** draft and **(b)** final *B. anynana* genome assemblies. Each contig/scaffold in the assembly is represented as a circle, coloured according to the best match to taxonomically annotated sequence databases (see legends) and distributed according to the proportion GC (*x*-axis) and read coverage (*y*-axis). The upper- and right-hand panels show the distribution of the total span (kb) of contigs/scaffolds for a given coverage (upper panel) or GC (right panel) bin. The heterozygosity in the sample is evident in the bimodal coverage distribution seen in (a). The cluster of orange-coloured contigs at a lower coverage and higher GC than the main cloud were likely derived from contaminant *Enterococcus* present in the sample. The final assembly, (b), shows the effective collapse of heterozygous regions, the removal of contaminant sequences and the scaffolding of contigs into long contiguous sequences. Note that only taxon annotations with a span > 1 Mb are shown in the legend for clarity.

Figure 3: Assembly and gene prediction comparison among 10 lepidopteran genomes. **(a)** Cumulative assembly curves showing the relationship between the number of scaffolds (*x*-axis) and the cumulative span of each assembly (*y*-axis), coloured by species. Higher quality assemblies are represented by an almost-vertical line (e.g., *H. melpomene* Hmel2 assembly in black), indicating a relatively small number of scaffolds is required to reach the final genome span; conversely, a long tail indicates the assembly includes a large number of smaller scaffolds. The curve for *B. anynana* (brown and bold) suggests a good assembly for this species, with the majority of the assembly comprised of relatively few scaffolds. **(b)** *B. anynana* v1.2 encodes the greatest number of genes of the 10 genomes, and is particularly

divergent from *B. mori*, which is of equivalent length. Species names/colours are as follows: "bicyclus" (brown), *B. anynana*; "bombyx" (blue), *B. mori*; "danaus" (light green), *D. plexippus*; "heliconius" (black), *H. melpomene*; "lerema" (dark green), *L. accius*; "melitaea" (orange), *M. cinxia*; "glaucus" (red), *P. glaucus*; "polytes" (pink), *P. polytes*; "xuthus" (violet), *P. xuthus*; "plutella" (grey), *P. xylostella*.

References

1. Brakefield PM, Beldade P, Zwaan BJ. The African butterfly *Bicyclus anynana*: a model for evolutionary genetics and evolutionary developmental biology. Cold Spring Harb Protoc. 2009; doi:10.1101/pdb.emo122–2.

2. Brakefield PM. Radiations of mycalesine butterflies and opening up their exploration of morphospace. Am. Nat. 2010;176 Suppl 1:S77–87.

3. Prudic KL, Jeon C, Cao H, Monteiro A. Developmental plasticity in sexual roles of butterfly species drives mutual sexual ornamentation. Science. 2011;331:73–5.

4. Westerman EL, Hodgins-Davis A, Dinwiddie A, Monteiro A. Biased learning affects mate choice in a butterfly. Proc. Natl. Acad. Sci. U.S.A. 2012;109:10948–53.

5. Monteiro A. Origin, development, and evolution of butterfly eyespots. Annu. Rev. Entomol. 2015;60:253–71.

6. Aduse-Poku K, Brakefield PM, Wahlberg N, Brattström O. Expanded molecular phylogeny of the genus *Bicyclus* (Lepidoptera: Nymphalidae) shows the importance

of increased sampling for detecting semi-cryptic species and highlights potentials for future studies. Systematics and Biodiversity. 2016; doi:10.1080/14772000.2016.1226979.

7. Brakefield PM, Reitsma N. Phenotypic plasticity, seasonal climate and the population biology of *Bicyclus* butterflies (Satyridae) in Malawi. Ecol Entomol. 1991;16:291–303.

8. Brakefield PM, Gates J, Keys D, Kesbeke F, Wijngaarden PJ, Monteiro A, et al. Development, plasticity and evolution of butterfly eyespot patterns. Nature. 1996;384:236–42.

9. Monteiro A, Tong X, Bear A, Liew SF, Bhardwaj S, Wasik BR, et al. Differential Expression of Ecdysone Receptor Leads to Variation in Phenotypic Plasticity across Serial Homologs. PLoS Genet. 2015;11:e1005529.

10. Beldade P, Mateus ARA, Keller RA. Evolution and molecular mechanisms of adaptive developmental plasticity. Mol Ecol. 2011;20:1347–63.

11. Oostra V, Brakefield PM, Hiltemann Y, Zwaan BJ, Brattström O. On the fate of seasonally plastic traits in a rainforest butterfly under relaxed selection. Ecol Evol. 2014;4:2654–67.

12. Dion E, Monteiro A, Yew JY. Phenotypic plasticity in sex pheromone production in *Bicyclus anynana* butterflies. Sci Rep. 2016;6:39002.

13. Jiang H, Lei R, Ding S-W, Zhu S. Skewer: a fast and accurate adapter trimmer for next-generation sequencing paired-end reads. BMC Bioinformatics. 2014;15:182.

14. Andrews S. FastQC: A quality control tool for high throughput sequence data. Available from: http://www.bioinformatics.babraham.ac.uk/projects/fastqc

15. Bushnell B. BBMap: BBMap short read aligner. Available from: sourceforge.net/projects/bbmap/

16. Saccheri IJ, Brakefield PM, Nichols RA. Severe inbreeding depression and rapid fitness rebound in the butterfly *Bicyclus anynana* (Satyridae). Evolution. 1996;50:2000–13.

17. Kumar S, Jones M, Koutsovoulos G, Clarke M, Blaxter M. Blobology: exploring raw genome data for contaminants, symbionts and parasites using taxon-annotated GC-coverage plots. Front Genet. 2013;4:237.

18. Altschul SF, Madden TL, Schaffer AA, Zhang JH, Zhang Z, Miller W, et al. Gapped BLAST and PSI-BLAST: a new generation of protein database search programs. Nucleic Acids Res. 1997;25:3389–402.

19. Buchfink B, Xie C, Huson DH. Fast and sensitive protein alignment using DIAMOND. Nature Methods. 2015;12:59–60.

20. Laetsch DR. Blobtools: Application for the visualisation of draft genome assemblies and general QC. Available from: https://github.com/DRL/blobtools

21. Kajitani R, Toshimoto K, Noguchi H, Toyoda A, Ogura Y, Okuno M, et al. Efficient de novo assembly of highly heterozygous genomes from whole-genome shotgun short reads. Genome Res. 2014;24:1384–95.

22. Boetzer M, Henkel CV, Jansen HJ, Butler D, Pirovano W. Scaffolding pre-

assembled contigs using SSPACE. Bioinformatics. 2011;27:578–9.

23. Xue W, Li J-T, Zhu Y-P, Hou G-Y, Kong X-F, Kuang Y-Y, et al. L_RNA_scaffolder: scaffolding genomes with transcripts. BMC Genomics. 2013;14:604.

24. Koutsovoulos G. SCUBAT2. Available from: https://github.com/GDKO/SCUBAT2

25. Haas BJ, Papanicolaou A, Yassour M, Grabherr M, Blood PD, Bowden J, et al. De novo transcript sequence reconstruction from RNA-seq using the Trinity platform for reference generation and analysis. Nat Protoc. 2013;8:1494–512.

26. Boetzer M, Pirovano W. SSPACE-LongRead: scaffolding bacterial draft genomes using long read sequence information. BMC Bioinformatics. 2014;15:211.

27. Boetzer M, Pirovano W. Toward almost closed genomes with GapFiller. Genome Biol. 2012;13:R56.

28. English AC, Richards S, Han Y, Wang M, Vee V, Qu J, et al. Mind the Gap: Upgrading Genomes with Pacific Biosciences RS Long-Read Sequencing Technology. PLoS ONE. 2012;7.

29. Li H, Durbin R. Fast and accurate long-read alignment with Burrows-Wheeler transform. Bioinformatics. 2010;26:589–95.

30. Dobin A, Davis CA, Schlesinger F, Drenkow J, Zaleski C, Jha S, et al. STAR: ultrafast universal RNA-seq aligner. Bioinformatics. 2013;29:15–21.

31. Parra G, Bradnam K, Korf I. CEGMA: a pipeline to accurately annotate core genes in eukaryotic genornes. Bioinformatics. 2007;23:1061–7.

32. Simão FA, Waterhouse RM, Ioannidis P, Kriventseva EV, Zdobnov EM. BUSCO: assessing genome assembly and annotation completeness with single-copy orthologs. Bioinformatics. 2015;31:3210–2.

33. Jurka J, Kapitonov VV, Pavlicek A, Klonowski P, Kohany O, Walichiewicz J. Repbase Update, a database of eukaryotic repetitive elements. Cytogenet Genome Res. 2005;110:462–7.

34. Smit A, Hubley R, Green P. RepeatMasker. Available from http://www.repeatmasker.org.

35. Koutsovoulos G. CGP-Pipeline. Available from: https://gist.github.com/GDKO

36. Holt C, Yandell M. MAKER2: an annotation pipeline and genome-database management tool for second-generation genome projects. BMC Bioinformatics. 2011;12:491.

37. Korf I. Gene finding in novel genomes. BMC Bioinformatics. 2004;5:59.

38. Ter-Hovhannisyan V, Lomsadze A, Chernoff YO, Borodovsky M. Gene prediction in novel fungal genomes using an ab initio algorithm with unsupervised training. Genome Res. 2008;18:1979–90.

39. Stanke M, Diekhans M, Baertsch R, Haussler D. Using native and syntenically mapped cDNA alignments to improve de novo gene finding. Bioinformatics. 2008;24:637–44.

40. Jones P, Binns D, Chang H-Y, Fraser M, Li W, McAnulla C, et al. InterProScan 5: genome-scale protein function classification. Bioinformatics. 2014;30:1236–40.

41. Challis RJ, Kumar S, Dasmahapatra KKK, Jiggins CD, Blaxter M. Lepbase: the Lepidopteran genome database. bioRxiv. 2016; doi: http://dx.doi.org/10.1101/056994

42. Duan J, Li R, Cheng D, Fan W, Zha X, Cheng T, et al. SilkDB v2.0: a platform for silkworm (*Bombyx mori*) genome biology. Nucleic Acids Res. 2010;38:D453–6.

43. Zhan S, Merlin C, Boore JL, Reppert SM. The monarch butterfly genome yields insights into long-distance migration. Cell. 2011;147:1171–85.

44. Heliconius Genome Consortium. Butterfly genome reveals promiscuous exchange of mimicry adaptations among species. Nature. 2012;487:94–8.

45. Davey JW, Chouteau M, Barker SL, Maroja L, Baxter SW, Simpson F, et al. Major improvements to the *Heliconius melpomene* genome assembly used to confirm 10 chromosome fusion events in 6 million years of butterfly evolution. G3. 2016;6:695–708.

46. Cong Q, Borek D, Otwinowski Z, Grishin NV. Skipper genome sheds light on unique phenotypic traits and phylogeny. BMC Genomics. 2015;16:639.

47. Ahola V, Lehtonen R, Somervuo P, Salmela L, Koskinen P, Rastas P, et al. The Glanville fritillary genome retains an ancient karyotype and reveals selective chromosomal fusions in Lepidoptera. Nature Communications. 2014;5:1–9.

48. Cong Q, Borek D, Otwinowski Z, Grishin NV. Tiger swallowtail genome reveals mechanisms for speciation and caterpillar chemical defense. Cell Rep. 2015;10:910–9.

49. Nishikawa H, Iijima T, Kajitani R, Yamaguchi J, Ando T, Suzuki Y, et al. A genetic mechanism for female-limited Batesian mimicry in *Papilio* butterfly. Nat Genet.

2015;47:405–9.

50. You M, Yue Z, He W, Yang X, Yang G, Xie M, et al. A heterozygous moth genome provides insights into herbivory and detoxification. Nat Genet. 2013;45:220–5.

51. Yates A, Akanni W, Amode MR, Barrell D, Billis K, Carvalho-Silva D, et al. Ensembl 2016. Nucleic Acids Res. 2016;44:D710–6.

 Click here to download Table Table1.docx

Tables

Table 1: Data counts and library information.

Library type	Platform	Read length	Insert (mean)	Number of reads (raw)	Number of reads (trimmed)	Number of bases (trimmed)	SRA run accessions
Short insert	Illumina HiSeq2500	125 bp paired-end	350 bp	271,808,057 pairs	267,241,712 (98.3%)	66,334,099,834 (97.6%)	ERR1102671-2, ERR1102675-6
Short insert	Illumina HiSeq2500	125 bp paired-end	550 bp	241,050,065 pairs	234,269,871 (97.2%)	57,913,474,128 (96.1%)	ERR1102673-4, ERR1102677-8
Mate pair	Illumina HiSeq2500	100 bp paired-end	3 kb	77,105,680 pairs	31,848,200 (41.3%)	5,758,856,502 (37.3%)	ERR1750945
Mate pair	Illumina MiSeq	100 bp paired-end	3 kb	5,641,764 pairs	2,170,610 (38.5%)	397,993,018 (35.3%)	ERR754051
Mate pair	Illumina HiSeq2500	100 bp paired-end	5 kb	77,614,870 pairs	45,676,725 (58.9%)	8,203,769,131 (52.8%)	ERR1750946
Mate pair	Illumina	100 bp	5 kb	7,939,601 pairs	4,734,000 (59.6%)	861,352,793 (54.2%)	ERR754052

	MiSeq						
Long read	PacBio P6	0.80-50 kb	10kb	1,388,796	1199064 (86.3%)	4,086,394,966	ERR1797559-74

 Click here to download Table Table2.docx

Table 2: Summary of *B. anynana* genome assembly and comparison to selected lepidopteran genomes.

	B. anynana	***B. mori***	***D. plexippus***	***H. melpomene***	***M. cinxia***
**Assembly version**	v1.2	ASM15162v1	v3	Hmel2	MelCinx1.0
**Span**	475.4 Mb	481.8 Mb	248.6 Mb	275.2 Mb	389.9 Mb
**Contigs**					
Number	23,699	10,682	10,682	3,100	48,180
N50[1]	78.7 kb	111 kb	111 kb	328.9 kb	14.1 kb
NumN50[2]	1,543	8,075	548	214	7,366
**Scaffolds**					
Number	10,800	43,379	5,397	795	8,261
N50	638.3 kb	4,008.4 kb	715.6 kb	2,102.7 kb	119.3 kb
NumN50	194	38	101	34	970
N90	99.3 kb	61.1 kb	160.5 kb	273.1 kb	29.6 kb
NumN90	909	258	366	176	3,396
Shortest / longest	201 b / 5 Mb	53 b / 16.2 Mb	300 b / 6.2 Mb	394 b / 9.4 Mb	1.5 kb / 668 kb
G+C content	36.5%	37.7%	31.6%	32.8%	32.6%

**NNNs**					
Span	5.8 Mb (1.2%)	50.1 Mb (10.4%)	6.7 Mb (2.7%)	986 kb (0.4%)	28.9 Mb (7.4%)
N50	1.4 kb	5.0 kb	2.5 kb	2.4 kb	1.4 kb
**CEGMA**[3] (*n* = 248)	**C:**81.1%; **D:**1.1; **F:**97.2%	**C:**76.6%; **F:**96.8%	**C:**90.3%; **F:**96%	**C:**88.7%; **F:**96.8%	NA
**BUSCO**[3] (*n* = 1,066)	**C:**98.3%; **D:**1%; **F:**99.2%	**C:**97.5%; **D:**0.5%; **F:**98.4%	**C:**97.4%; **D:**8.6%; **F:**98.5%	**C:**98.8%; **D:**0.7%; **F:**99.3%	**C:**85.7%; **D:**0.2%; **F:**91.8%

[1]N50: the length of the contig/scaffold at which 50% of the genome span is accounted, given a list of sequences sorted by length. [2]numN50: the number of sequences required to reach the N50 sequence. [3]CEGMA / BUSCO notation: **C**, proportion (%) genes completely recovered; **D**, duplication rate; **F**, proportion (%) genes at least partially recovered (including complete genes); ***n***, number of queries. Note that duplication rate (D) for CEGMA is given as the average number of (complete) genes recovered, whereas for BUSCO it is the proportion of complete genes recovered multiple times. BUSCO values are based on comparisons to the Arthropoda gene set.

Click here to download Table Table5.docx

Table 5: Summary of *B. anynana* gene prediction.

	B. anynana	***B. mori***	***D. plexippus***	***H. melpomene***	***M. cinxia***
**Assembly version**	v1.2	ASM15162v1	v3	Hmel2	MelCinx1.0
**Number of genes**	22642	15488	15130	15261	16751
**Number of CDS**	22642	19618	15130	13178	16668
Mean length	1.4 kb	1.6 kb	1.4 kb	1.3 kb	958 b
Median length	1.2 kb	1.2 kb	981 b	927 b	693 b
Min/max	84 b / 28.3 kb	23 b / 60.3 kb	9 b / 58.9 kb	45 b / 46.4 kb	6 b / 45.4 kb
**Introns**					
Mean number per gene	4.4	9.9	5.7	5	NA[1]
Length (mean/median)	1.3/0.6 kb	2.4/0.8 kb	795/280 b	960/416 b	NA
**Exons**					
Length (mean/median)	208/126 b	283/161 b	206/149 b	284/157 b	NA
**Number of single-exon genes**	3571	1744	1461	3113	NA
**Transcript GC**	49.2%	48.3%	46.5%	43%	41.7%
**Gene frequency[2] (genes per Mb)**	47.7	32.1	60.9	55.5	NA

[1]GFF for *M. cinxia* not available; [2]Defined as the number of genes divided by the total genome span (Mb).

Figure 1

Click here to download Figure Fig1_g5848.pdf

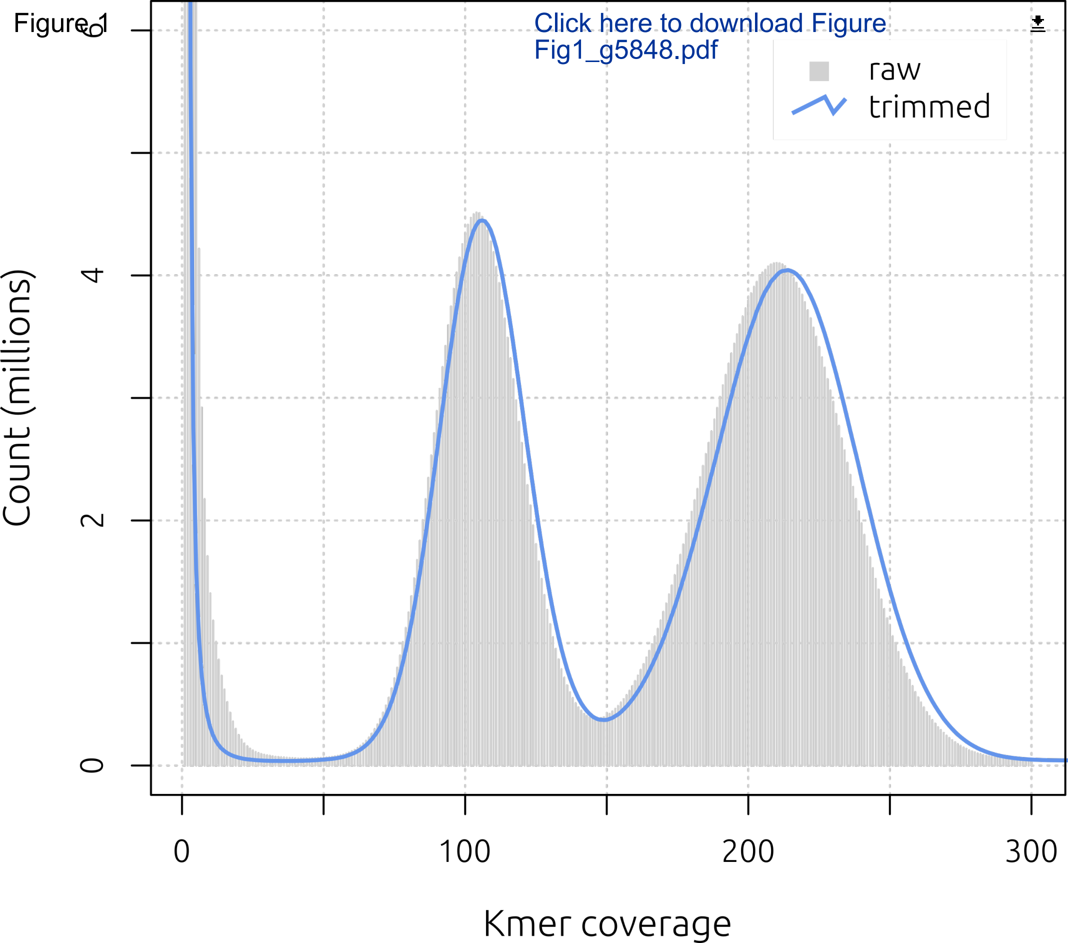

Figure 2

Click here to download Figure Fig2_g4698.pdf

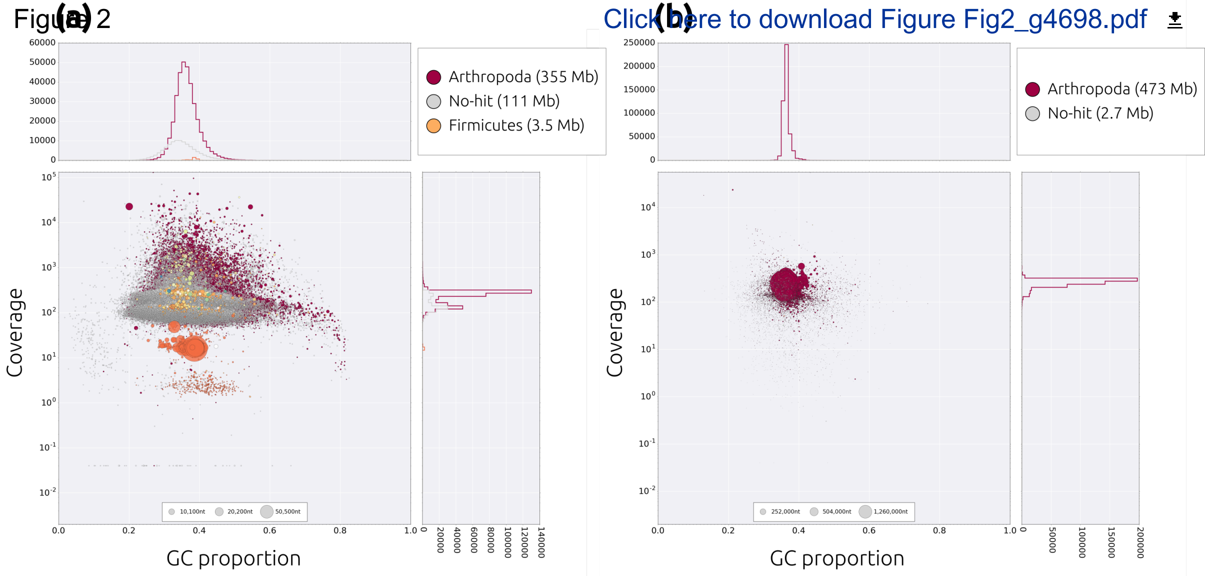

Figure 3

Click here to download Figure Fig3_g5525.pdf

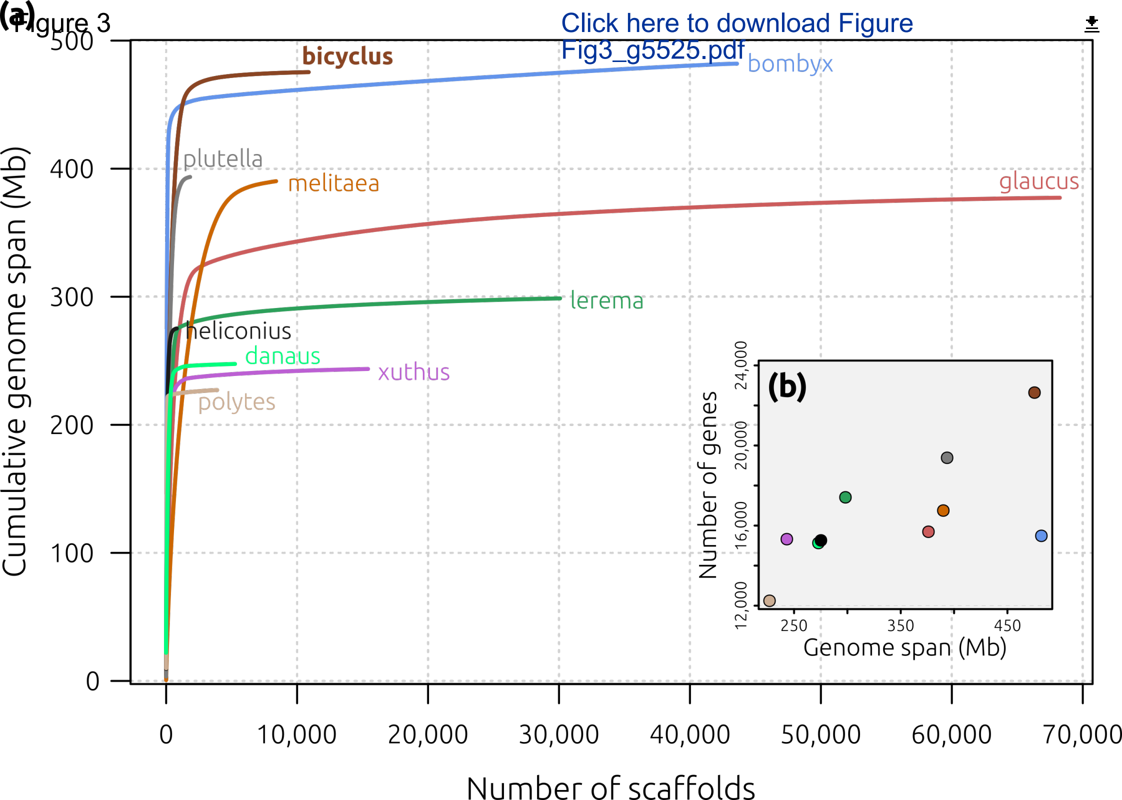

Supplement: GIGA-D-17-00016_Original_Submission.pdf [file gix035_GIGA-D-17-00016_Original_Submission.pdf]
