# Supplementary material for: A high-coverage draft genome of the mycalesine butterfly Bicyclus anynana
Source: Gigascience. 2017 May 9;6(7):1–7. doi: 10.1093/gigascience/gix035 (PMC5493746; doi:10.1093/gigascience/gix035)
Supplement: Response_to_Reviewers_Original_Submission.pdf [file gix035_Response_to_Reviewers_Original_Submission.pdf]

Reviewer #1: Nowell et al present their manuscript describing the generation of a high-coverage and relatively high-quality draft genome of the butterfly, Bicyclus anynana. The sequencing data included both Illumina and PacBio, which were combined (also transcriptome data) to build the assembly, with appropriate contaminant filtering steps. Genome annotation produced a larger-than-average gene set, but the retained models do appear to be well supported. The data and methods descriptions are clear and concise, and the supporting evidence is convincing. I agree that this draft genome sequence for B. anynana will serve as a key genomics resource for current and future studies of this important model species. Minor points:

- P9, line 160: 'smaller than the longest', perhaps 'smaller than the largest' or 'shorter than the longest'
- Response: this has been corrected to “smaller than the largest”.

- P9, line 164: 'that' => 'than'
- Response: this has been corrected.

- P13, line 247: 'divergent', perhaps 'different'
- Response: we have changed “divergent” to “different”.

- Table 5: please check - it seems that there are fewer CDSs than genes for Hmel2 and MelCinx1.0, unless I have misunderstood this could be an error as protein-coding genes can have one or more CDS, so surely the numbers of CDSs should always be greater than or equal to the number of (protein-coding) genes?
- Response: for some species, the “number of genes” (as given by LepBase) includes non-protein-coding loci such as tRNA genes etc. These are not predicted in all species, however; it depends on whether the research group responsible for each genome has included non-coding gene predictions in the GFF submitted to LepBase. Thus for some species the number of genes may be greater than the number of CDSs, whereas for other species the number of genes is equal and equivalent to the number of CDSs. We agree this is a bit confusing and have decided to remove the row entitled “Number of genes” from Table 5, leaving “Number of CDS”, which can be read less ambiguously as “the number of protein coding genes”.

Reviewer #2: The paper of Nowell et al is a typical well-made genome paper, presenting a good quality genome of a Lepidopteran insect. The sequencing, assembly, annotation and quality assessment are very well performed. The genome data and features are already accessible through a public dedicated database. I thus have no major issues against its publication in GigaScience. I only have minor interrogations below:

- When describing the different libraries, I did not understand whether the insert sizes indicated in the tables and text are the expected ones, or the observed data.
- Response: the insert sizes given in Table 1 are the expected insert sizes. This has been clarified by the addition of the word “expected” in the relevant column header in Table 1.

- Concerning the discarding of some reads as putative contaminants, did you check their GC content? More generally, did you scan the reads or contigs for GC content to may be identify "outgroups" that might correspond to putative contaminants? And what could be the nature of these contaminants? May be they correspond to microorganisms associated with B. anynana. Or correspond to mitochondrial genome?
- Response: the %GC of all contigs from the initial draft genome is shown in Fig. 2a. This includes contigs which were then marked as “putative contaminants” based on %GC and/or taxonomic classification; all reads which mapped to these contigs were then discarded prior to the final assembly. Thus the %GC of contaminant reads is represented by the %GC of the contaminant contigs these reads have been assembled into, which are visualised in Fig. 2a. The taxonomic distribution of inferred contaminants was biased towards numerous bacterial groups, including a well-assembled complete genome identified as the firmicute Enterococcus faecium, most likely a laboratory contaminant. We did not perform a thorough analysis of the nature of all identified contaminants, e.g., if they were possible co-symbionts of B. anynana in the wild, although we expect that most sequences from non-target organisms are contaminants. The B. anynana mt genome, as identified by high similarity to other lepidopteran whole mt genomes, is present in the assembly fasta file (scaffold ‘BANY01621’, 13.7 kb, 21.2% G+C).

- The authors clearly show that B. anynana has more predicted genes than other Lepidopteran genomes. The authors suggest a correlation with genome size (which is probably not a good explanation) as well as a consequence of high plasticity. This could be plausible: in the case of Daphnia and aphids, the high number of predicted genes was also discussed in regard to the capacity of these organisms to be plastic (polyphenism). However, those genomes are also characterized by many gene duplications or even expansions that could strengthen the hypothesis of neo-functionalization required for plasticity. Anything in that sense observed for B. anynana?
- Response: the genomic basis for polyphenism in B. anynana is an ongoing research question to which the genome sequence reported here will contribute. However, we chose to focus the current manuscript on reporting only the assembly and annotation procedures used to generate the genome sequence, and thus have not performed the necessary analyses to be able to answer this question here (e.g., a clustering analysis of orthologous genes across the Lepidoptera, to uncover potential gene-family expansions that may be unique to the Bicyclus lineage). We will be sure to bear your helpful observations in mind in future analyses!
