# Supplementary material for: A high-coverage draft genome of the mycalesine butterfly Bicyclus anynana
Source: Gigascience. 2017 May 9;6(7):1–7. doi: 10.1093/gigascience/gix035 (PMC5493746; doi:10.1093/gigascience/gix035)
Supplement: Reviewer_1_Report_(Original_Submission).pdf [file gix035_Reviewer_1_Report_(Original_Submission).pdf]

Reviewer Report

Title: "A high-coverage draft genome of the mycalesine butterfly Bicyclus anynana"

Version: Original Submission **Date:** 1/24/2017

Reviewer name: Evgeny Zdobnov

Reviewer Comments to Author:

Nowell et al present their manuscript describing the generation of a high-coverage and relatively high-quality draft genome of the butterfly, Bicyclus anynana. The sequencing data included both Illumina and PacBio, which were combined (also transcriptome data) to build the assembly, with appropriate contaminant filtering steps. Genome annotation produced a larger-than-average gene set, but the retained models do appear to be well supported. The data and methods descriptions are clear and concise, and the supporting evidence is convincing. I agree that this draft genome sequence for B. anynana will serve as a key genomics resource for current and future studies of this important model species.Minor points:P9, line 160: 'smaller than the longest', perhaps 'smaller than the largest' or 'shorter than the longest'P9, line 164: 'that' => 'than'P13, line 247: 'divergent', perhaps 'different' Table 5: please check - it seems that there are fewer CDSs than genes for Hmel2 and MelCinx1.0, unless I have misunderstood this could be an error as protein-coding genes can have one or more CDS, so surely the numbers of CDSs should always be greater than or equal to the number of (protein-coding) genes?

Level of Interest

Please indicate how interesting you found the manuscript: An article whose findings are important to those with closely related research interests

Quality of Written English

Please indicate the quality of language in the manuscript: Acceptable

Declaration of Competing Interests

Please complete a declaration of competing interests, considering the following questions:

- Have you in the past five years received reimbursements, fees, funding, or salary from an organisation that may in any way gain or lose financially from the publication of this manuscript, either now or in the future?
- Do you hold any stocks or shares in an organisation that may in any way gain or lose financially from the publication of this manuscript, either now or in the future?
- Do you hold or are you currently applying for any patents relating to the content of the manuscript?
- Have you received reimbursements, fees, funding, or salary from an organization that holds or has applied for patents relating to the content of the manuscript?
- Do you have any other financial competing interests?
- Do you have any non-financial competing interests in relation to this paper?

If you can answer no to all of the above, write 'I declare that I have no competing interests' below. If your reply is yes to any, please give details below.

I declare that I have no competing interests

I agree to the open peer review policy of the journal. I understand that my name will be included on my report to the authors and, if the manuscript is accepted for publication, my named report including any attachments I upload will be posted on the website along with the authors' responses. I agree for my report to be made available under an Open Access Creative Commons CC-BY license (http://creativecommons.org/licenses/by/4.0/). I understand that any comments which I do not wish to be included in my named report can be included as confidential comments to the editors, which will not be published.

I agree to the open peer review policy of the journal
