# Supplementary material for: A high-coverage draft genome of the mycalesine butterfly Bicyclus anynana
Source: Gigascience. 2017 May 9;6(7):1–7. doi: 10.1093/gigascience/gix035 (PMC5493746; doi:10.1093/gigascience/gix035)
Supplement: Reviewer_2_Report_(Original_Submission).pdf [file gix035_Reviewer_2_Report_(Original_Submission).pdf]

Reviewer Report

Title: "A high-coverage draft genome of the mycalesine butterfly Bicyclus anynana"

Version: Original Submission **Date:** 2/3/2017

Reviewer name: Denis Tagu

Reviewer Comments to Author:

The paper of Nowel et al is a typical well-made genome paper, presenting a good quality genome of a Lepidopteran insect. The sequencing, assembly, annotation and quality assessment are very well performed. The genome data and features are already accessible thought a public dedicated database. I thus have not major issues against its publication in GigaScience.I only have minor interrogations below:When describing the different libraries, I did not understand whether the insert sizes indicated in the tables and text are the expected ones, or the observed data.Concerning the discarding of some reads as putative contaminants, did you check their GC content? More generally, did you scan the reads or contigs for GC content to may be identify "outgroups" that might correspond to putative contaminants? And what could be the nature of these contaminants? May be they correspond to microorganisms associated with B. anynana. Or correspond to mitochondrial genome?The authors clearly show that B. anynana has more predicted genes than other Lepidopteran genomes. The authors suggest a correlation with genome size (which is probably not a good explanation) as well as a consequence of high plasticity. This could be plausible: in the case of Daphnia and aphids, the high number of predicted genes was also discussed in regard to the capacity of these organisms to be plastic (polyphenism). However, those genomes are also characterized by many gene duplications or even expansions that could strengthen the hypothesis of neo-functionalization required for plasticity. Anything in that sense observed for B. anynana?

Level of Interest

Please indicate how interesting you found the manuscript: An article whose findings are important to those with closely related research interests

Quality of Written English

Please indicate the quality of language in the manuscript: Acceptable

Declaration of Competing Interests

Please complete a declaration of competing interests, considering the following questions:

- Have you in the past five years received reimbursements, fees, funding, or salary from an organisation that may in any way gain or lose financially from the publication of this manuscript, either now or in the future?

- Do you hold any stocks or shares in an organisation that may in any way gain or lose financially from the publication of this manuscript, either now or in the future?
- Do you hold or are you currently applying for any patents relating to the content of the manuscript?
- Have you received reimbursements, fees, funding, or salary from an organization that holds or has applied for patents relating to the content of the manuscript?
- Do you have any other financial competing interests?
- Do you have any non-financial competing interests in relation to this paper?

If you can answer no to all of the above, write 'I declare that I have no competing interests' below. If your reply is yes to any, please give details below.

I declare that I have no competing interests

I agree to the open peer review policy of the journal. I understand that my name will be included on my report to the authors and, if the manuscript is accepted for publication, my named report including any attachments I upload will be posted on the website along with the authors' responses. I agree for my report to be made available under an Open Access Creative Commons CC-BY license (http://creativecommons.org/licenses/by/4.0/). I understand that any comments which I do not wish to be included in my named report can be included as confidential comments to the editors, which will not be published.

I agree to the open peer review policy of the journal
